# The Early Treatment of Burns and Scalds

**Published:** 1880-03

**Authors:** E. Fletcher Ingals

**Affiliations:** Lecturer on Physical Diagnosis and Diseases of the Chest, Rush Medical College. Clinical Professor of Diseases of the Throat and Chest, Woman’s Medical College; 188 Clark St., Chicago


### ARTICLE II.

### THE EARLY TREATMENT OF BURNS AND SCALDS.

E. FLETCHER INGALS, M.D., Lecturer on Physical Diagnosis and Diseases of the Chest, Rush Medical College. Clinical Professor of Diseases of the Throat and Chest, Woman's Medical College.

On the 24th of October last, I was summoned by telephone to see Mr. L—— A——, a vigorous man forty-nine years of age, who had just met with a severe accident.

I met the patient when he was brought home, about three-quarters of an hour after having been severely scalded.

In passing through the factory he had stepped on the cover of a catch-basin, which received the exhaust steam from the boiler; the cover suddenly gave way, and he was percipitated into the boiling water beneath, which covered the feet and legs nearly to the knees. The legs must have remained in the water for several seconds, and the patient thought they were immersed a second time before he could extricate himself from his painful position. The man's cries brought help, and he was carried into the office, the shoes removed, and cotton dressings saturated in carron oil, applied to the burns.

While the patient was being brought into his house, he was taken with a severe rigor, for which a large dose of whisky was administered. I examined the legs and found them burned from about an inch above the soles of the feet, to within three or four inches of the knees, making a scald on each leg a foot in length, and encircling the entire member. The tissues were burned most deeply just above the ankle joint.

The rigor continued notwithstanding the whisky. I at once ordered a tub of cold water, in which the patient's feet and legs were immersed with the effect of instantly relieving the pain, and at once overcoming the chill. The water was used just as drawn from the hydrant, and stood at 67° F. I found that if the temperature of the water rose only a few degrees, the burning pain would return, therefore I ordered that the temperature be kept at 67°, either by means of pieces of ice or by changing the water.

I gave no more whisky and no anodyne as none was needed. I directed the patient to keep the legs constantly in the water for six or seven hours, and longer if necessary, to prevent the return of pain, and that the temperature should be gradually increased but always kept low enough to prevent smarting.

When I returned in the evening, I found the patient perfectly comfortable, with his feet still in the water. He had removed them once or twice for a few minutes, but as soon as smarting returned they had been again immersed.

I now wrapped the legs in oiled cotton and put the patient in bed, but left directions for him to again put the feet in water if pain returned. The next morning, I found that the patient had remained about an hour in bed, when it became necessary to put the feet again in water. Subsequently the cold water treatment was continued with only slight intermissions until it had been used altogether for seventy-four hours, at which time it became unnecessary as the pain did not return. The temperature of the water had been gradually increased until during the latter part f the treatment, when it stood at nearly 100°.

In instituting this treatment, I was guided by personal experience in the case of a small but deep burn, and by the well-known fact that cold water will relieve the pain of burns and that such injuries if of any considerable extent are very likely to prove fatal in consequence of constitutional disturbance, caused by the pain and inflammation.

The question in my own mind as to the injurious effects of such long immersion in cold water, was answered by reference to the experience of Captain Webb, who spent eighteen hours in the English Channel without apparent detriment to his health.

Quite an extensive experience in the care of burns, in the hospital, after the fire in 1871 and the subsequent care of several scalds, convinced me long ago that the proper course to follow in recent burns or scalds was the one which I pursued in this case. I have frequently suggested the method to other physicians, but have always been met by reference to the well-known dressings of lead, carron oil, bismuth, etc., etc., all of which have long been found useful, but none of which will stop the pain, or so limit the inflammation as the popular and easily accessible remedy, which was used in this case.

When the use of cold water became unnecessary in this case, the legs were dressed with white lead and cotton, which was kept on until suppuration occurred; subsequently the separation of sloughs was favored by poultices and in sixteen or seventeen days from the date of the injury the dead skin had all been removed and the ulcers presented a clean and healthy appearance.

I found that the skin had been burned in varying degrees from near the sole of the foot to the upper part of the calf of the leg; in some places only the outer layer having been destroyed, while just above the ankle on either leg, a strip of skin, varying from two and one-half to five inches in width, had been destroyed throughout its entire thickness.

The effects of the cold water were now very marked in the evident limitation of the inflammation. In many places from one-fourth to three-fourths of the entire thickness of the true skin had been destroyed and the lower layers remained intact. In still other places, nearly the whole thickness of the skin had been destroyed, leaving here and there, small points of the deeper layers, one or two lines in diameter. I have never seen such small portions of skin saved by any of the ordinary methods of treatment, under which, it seems to me, the tendency of inflammation to involve the entire thickness of the skin, would certainly have been fatal to these. From all these points new skin was rapidly formed, so that in a short time the greater portion of the sores was healed.

The only unfavorable symptoms during this time were a few intermittent chills and fever, which were readily checked by quinine; nervousness, which prevented good sleep, for the first two weeks, and occasional, irregular pains in different parts of the legs. However, the patient never lost a meal and constantly improved in flesh throughout his confinement to the house.

About the end of the fourth week several small grafts of skin were inserted in the ulcers and Martin's rubber bandages applied. Skin-grafting was subseqently practiced several times, but only five or six of the points grew; caustics were used occasionally toward the latter part of the treatment to cut down exuberant granulations which were not wholly controlled by the bandage. The bandage was kept on the legs until recovery was complete and is still worn for support during the day. The patient began to walk about in six or seven weeks and was out of the house December 27th and the case was completed a few days later.

From my experience with this and other methods of treating scalds and burns, I feel confident that the cold water dressing is the best in all cases where it can be applied soon after the injury has been received and probably it would be best in all cases if applied any time after the pain is felt, and continued constantly, with gradual elevation of the temperature, until it ceases.

This treatment will stop the pain and prevent the constitutional disturbance which it would occasion, thus enabling the physician to save the lives of many patients, who would otherwise die of shock.

It will limit the resulting inflammation to the minimum degree and thus preserve the greatest possible amount of tissue.

If the temperature of the water is gradually elevated as the burning pain disappears, and the treatment is discontinued when this has been accomplished, no evil results need be feared, but the physician may have the satisfaction of giving his patient the greatest possible relief, at the same time preventing further destruction of tissue and probably restoring much that has already been injured.

The treatment must be continuous in order to be productive of the best results; probably it would be hurtful in some cases if used intermittently.

The laity should be instructed, in cases of burns or scalds from burning clothing, hot water, steam, etc., to immediately immerse the parts in water cold enough to relieve the pain and to keep them in that condition until the smarting or burning would not recur and until a physician could be summoned.

If this plan were adopted there is good reason to hope that many of the extensive, but superficial burns, which usually prove fatal, might be transformed into slight injuries, and many of the ugly deformities which originate in these accidents might be prevented.

188 Clark St., Chicago.

